# MIND‐China in 2018‐2023: Multimodal interventions in rural communities

**DOI:** 10.1002/alz.086888

**Published:** 2025-01-09

**Authors:** Chengxuan Qiu

**Affiliations:** ^1^ Aging Research Center, Karolinska Institutet and Stockholm University, Stockholm Sweden

## Abstract

**Background:**

Worldwide, ∽40% of dementia cases are preventable by interventions to target major modifiable risk factors. In the multimodal interventions to delay dementia and disability in rural China (MIND‐China), we aim to test the effect of multimodal intervention programs on maintaining cognitive and physical function among rural‐dwelling older adults and discuss about challenges and opportunities for a multidomain intervention study in a rural population.

**Method:**

MIND‐China targets people who are aged 60‐79 years and living in rural communities (52 villages) in Shandong. The study tested two intervention programs: The vascular intervention included treatment of hypertension, diabetes, and dyslipidemia; the multimodal intervention included vascular interventions plus non‐pharmacological intervention (e.g., group exercise, personalized leisure activities, and cognitive training). The control group received regular primary care. Baseline assessments and recruitment for interventions were performed in 2018‐2019. Follow‐up assessments were conducted in 2021 and 2023. The primary outcome is the composite cognitive z‐score from a neuropsychological test battery. Secondary outcomes are physical function, dementia, cardiovascular events, and mortality. Measures were taken to overcome barriers for study participation and to achieve high rate of participating interventions and follow‐up assessments.

**Result:**

At baseline (March‐September 2018), 5765 persons (74.9% of all eligible persons; age ≥60 years; 57.2% women; 40.6% illiteracy; and 88.3% farmers) were recruited. In 2019, recruitment for interventions was performed aiming to recruiting 3000 participants who were aged 60‐79 years and free of dementia and disability. The cluster(village)‐based randomization method was used to assign participants into three groups. The intervention programs were organized by local coordinators and village doctors, even during COVID‐19 pandemic, whenever possible. Follow‐up assessments for intervention participants were conducted in 2021 and 2023. The interventions ended in October‐November 2023. The assessments and diagnosis of outcomes and data cleaning are ongoing. Factors associated with participation in and adherence to the interventions and follow‐up assessment are being analyzed.

**Conclusion:**

MIND‐China successfully recruited a large sample of participants for a multimodal intervention in rural communities. The project is expected to clarify whether the personalized culture‐sensitive multimodal interventions may help maintain cognitive and physical function among rural elderly populations in China.

